# Cardiorespiratory fitness and the incidence of type 2 diabetes: a cohort study of Japanese male athletes

**DOI:** 10.1186/1471-2458-14-493

**Published:** 2014-05-23

**Authors:** Yuki Someya, Sachio Kawai, Yoshimitsu Kohmura, Kazuhiro Aoki, Hiroyuki Daida

**Affiliations:** 1 School of Health and Sports Science, Juntendo University, 1-1 Hiraga-gakuenndai, Inzai, Chiba 270-1695, Japan; 2 Department of Cardiology, Juntendo University, 2-1-1 Hongo, Bunkyo-ku, Tokyo 113-8421, Japan

**Keywords:** Cardiorespiratory fitness, Type 2 diabetes, Japanese, Athletes, Cohort study

## Abstract

**Background:**

In Japan, although the incidence of overweight (BMI ≥ 25) is still low compared with that in Europe and the United States, the prevalence of type 2 diabetes has increased over the last 15 years,. In both Japanese and Caucasian populations it has been reported that a high level of cardiorespiratory fitness protects against the development of type 2 diabetes. However, there are no reports focused specifically on athletes that investigate whether high cardiorespiratory fitness at a young age can prevent disease later in life. We examined the relationship between cardiorespiratory fitness at a young age and the development of type 2 diabetes in Japanese athletes using a cohort study.

**Methods:**

The cardiorespiratory fitness of male alumni of the physical education department of Juntendo University, as measured by stored data of a 1,500-m endurance run in college (1971–1991) was compared with their incidence of type 2 diabetes as determined by follow-up questionnaires (2007–2009). This study used Cox’s proportional hazards models and adjusted for age, year of graduation, BMI, smoking, and sports club participation at college age.

**Results:**

We collected data on cardiorespiratory fitness at college age and medical history survey data during 2007–2009 from 570 male alumni. The median follow-up period was 26 years (IQR: 23–29 years), and 22 men had developed type 2 diabetes. An inverse relationship was observed between incidence of type 2 diabetes and level of cardiorespiratory fitness at time of college after adjustment for age, year of graduation, BMI, smoking, and sports participation. The adjusted hazards ratio and 95% CI by category (low, medium, and high) were 1.00 (reference), 0.40 (0.14–1.13) and 0.26 (0.07–1.00) (p = 0.03 for trend).

**Conclusions:**

A high level of cardiorespiratory fitness at a young age can help prevent type 2 diabetes later in life.

## Background

Worldwide, more than 371 million people over age 20 have type 2 diabetes [1]. In Japan, although the prevalence of overweight (BMI ≥ 25), the strongest predisposing factor for type 2 diabetes, is low compared with that in Europe and the United States, the prevalence of type 2 diabetes has increased [2]. The National Health and Nutrition Survey reported that type 2 diabetes incidence in Japan increased by 30% between 1997 and 2007 [2,3]. In addition, the International Diabetes Federation reported in 2012 that Japan had the ninth-highest rate of type 2 diabetes in the world [1]. In epidemiological studies conducted in Western countries, a high level of cardiorespiratory fitness has been shown to be a protective factor against type 2 diabetes [4-6]. In Japan, few prospective epidemiological studies have investigated the relationship between cardiorespiratory fitness and type 2 diabetes. Sawada et al. showed that a low level of cardiorespiratory fitness in middle age is a strong risk factor for type 2 diabetes [7].

Few epidemiological studies have investigated the relationship between cardiorespiratory fitness and type 2 diabetes in athletes. It has been reported that former top-level athletes, and particularly male former endurance athletes, have a lower prevalence of type 2 diabetes [8-11]. It has also been reported former that top-level athletes, such as weightlifters, boxers, and track and field throwers, have an equal or higher prevalence of type 2 diabetes compared with healthy men who are not athletes [8,9,11]. Although top athletes are a biologically selected group given particular training, maintaining high cardiorespiratory fitness levels may directly contribute to the prevention of type 2 diabetes. However, in these studies, former top athletes were grouped according to the type of sports they played rather than according to measured data of their cardiorespiratory fitness levels. As these studies [8,9,11] have been carried out only in Caucasian populations, it was not clear whether a high level of cardiorespiratory fitness at a young age can prevent the incidence of type 2 diabetes in Asian athletes, who are generally not overweight. Therefore, the present study examined the relationship between cardiorespiratory fitness at a young age and the development of type 2 diabetes in Japanese athletes using a cohort study.

## Methods

### Subjects

**Figure 1:**
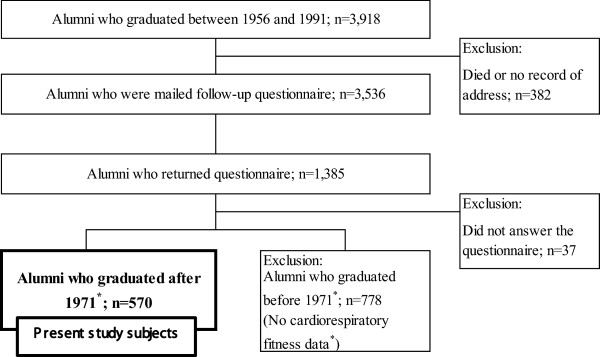
Flowchart of subjects in this study.

This study included male alumni of the Department of Physical Education of Juntendo University. Students were selected for admission to college by an entrance examination of motor skills and other tests. They came from all over Japan. Almost all students were members of a college sports club, such as track and field, gymnastics, soccer, or judo and they participated in training in these areas. In this college, an athletic test included an annual test of cardiorespiratory fitness, and these data were available for the years 1971 and thereafter. Between 2007 and 2009, 3,539 male alumni who graduated between 1956 and 1991 answered a follow-up questionnaire about their medical background. Those who had died or for whom no record of address could be found were excluded. Female alumni were not included in this study because this university has only admitted them since 1991. Along with the questionnaire, prospective participants were sent a letter of informed consent approving the collection and use of their athletic test data for research purposes. Responses with signed and answered questionnaires were regarded as giving consent. Privacy precautions were maintained through Juntendo University, and all data were anonymized before analysis. The study protocol was reviewed and approved by the Juntendo University ethics committee in 2007. In the present study, we selected subjects from alumni whose cardiorespiratory fitness data at the time of college was available, and who responded to the follow-up questionnaire (Figure 1). Therefore, the subjects of this study were male alumni who graduated between 1971 and 1991.

### Cardiorespiratory fitness test

In this college, all alumni underwent an athletic test that included a test of cardiorespiratory fitness once a year. The test measured cardiorespiratory fitness using a 1,500-m endurance run. This study used the data from the subject’s last fitness test. At the time of the test, the body height and weight of the subjects were measured. We calculated the subjects’ body mass index (BMI: kg/m^2^) from these data.

### Investigation of diabetes prevalence

Between 2007 and 2009, a follow-up self-administered questionnaire was sent by mail to alumni. The questionnaire asked participants whether they had been diagnosed with type 2 diabetes by a physician after graduating from college. Those who had been diagnosed with type 2 diabetes were asked to indicate their age at diagnosis. Participants were also asked about smoking habits. We estimated smoking habits at the time of college from the answer.

### Statistical analysis

Thirty-nine percent of subjects, or 1,385 male alumni, returned the follow-up questionnaire. Cardiorespiratory fitness test data at the time of college were available for 41% of the respondents. In the present study, we analyzed 570 male alumni for whom cardiorespiratory fitness data were available, and who responded to the questionnaire (Figure 1). For each subject, the duration of follow-up was counted from the year of graduation (1971–1991) until the time of the follow-up questionnaire filled out between 2007 and 2009. Subjects’ cardiorespiratory fitness levels were categorized into tertiles (low, medium, and high) based on their 1,500-m endurance run at the time of college. The association between cardiorespiratory fitness at the time of college and the incidence of type 2 diabetes was assessed by Cox proportional hazards models. Data were adjusted for age, year of graduation, BMI, smoking, and college sports club participation. Multivariable-adjusted hazards ratios for type 2 diabetes and 95 percent confidence interval (95% CI) were obtained using the low cardiorespiratory fitness group as the reference category. All statistical analyses were conducted using SPSS 18.0 for Windows (SPSS Inc., Chicago, IL).

## Results

This study covered a 26-year follow-up period (IQR: 23–29 years) that included 14,576 person-years of observation. The median age at the time of the follow-up questionnaire was 49 years (IQR: 45–52 years). At follow-up, 22 men had developed type 2 diabetes. The median age and BMI of subjects at time of college was 23 years and 22.1 kg/m^2^. Ninety-nine percent of subjects had participated in a college sports club. The 1,500-m endurance run time was 325 seconds and the time taken ranged among subjects from 310 to 345 seconds (Table 1). Table 2 shows the physical characteristics of subjects at college stratified by cardiorespiratory fitness levels. Men in the highest cardiorespiratory fitness group had the lowest BMI levels and the lowest prevalence of smoking.

**Table 1:** Characteristic of all subjects

	**All**
Number of subjects	570
Age at follow up questionnaire	49 (45–52)
follow-up period	26 (23–29)
Person-years follow-up	14,576
Diagnosed type 2 diabetes^#^	22
1,500 meter endurance run time (seconds)^*^	325 (310–345)
Age (years)^*^	23 (23–23)
Year of graduation^*^	1982 (1979–1985)
BMI (kg/m^2^)^*^	22.1 (21-23.0)
Smoker (n,%)^*^	275 (48.2)
College sports club participation (n,%)^*^	563 (98.8)

Estimates show as median (IQR: interquartile Range) or number (%).

The rate smoking and college sports club participation counted “yes”.

*All items were the data at colleg age.

#Diabeters was diagnosed by physician.

**Table 2:** **Characteristics of male subjects by cardiorespiratory fitness level** (**tertile**)

	**Cardiorespiratory fitness level tertiles**		
	**Low**	**Medium**	**High**
Number of subjects	189	186	195
1,500 meter endurance run time (seconds)	353 (345–365)	325 (320–330)	301 (290–310)
Age (years)	23 (23–23)	23 (23–23)	23 (23–23)
Year of graduation	1982 (1978–1985)	1981 (1978–1985)	1983 (1979–1986)
BMI (kg/m^2^)	22.5 (21.4-23.7)	22.3 (21.3-23.4)	21.4 (20.5-22.3)
Smoker^*^ (n,%)	111 (58.7)	106 (57.0)	58 (29.7)
College sports club participation^*^ (n,%)	185 (97.9)	183 (98.4)	195 (100.0)

Estimates shown as median (IQR: interquartile Range) or number (%).

All items were the data at colleg age.

*The rate smoking and college sports club participation counted “yes”.

Table 3 shows the relationship between potential risk factors and type 2 diabetes risk estimated by the Cox proportional hazards model. Potential risk factors including age, year of graduation, BMI, smoking, and college sports club participation at the time of college showed no relationship with type 2 diabetes risk in this study.

**Table 3:** Potential risk factors for diabetes

	Multivariable adjusted hazard ratio^*^	**(95% CI)**
Age (years)	0.56	(0.27–1.15)
Year of graduation	0.67	(0.32–1.39)
BMI (kg/m^2^)	1.17	(0.99–1.38)
Smoker (yes/no)	0.59	(0.23–1.50)
College sports club participation (yes/no)	0.80	(0.06–10.16)

Estimates shown as median (IQR) or number (%) and Multivariable adjusted hazard ratio (95% CI).

All items refer to the data of college age subjects.

*Cox proportional hazards models adjusted for 1,500-m endurance run time and all items in the table.

**Figure 2:**
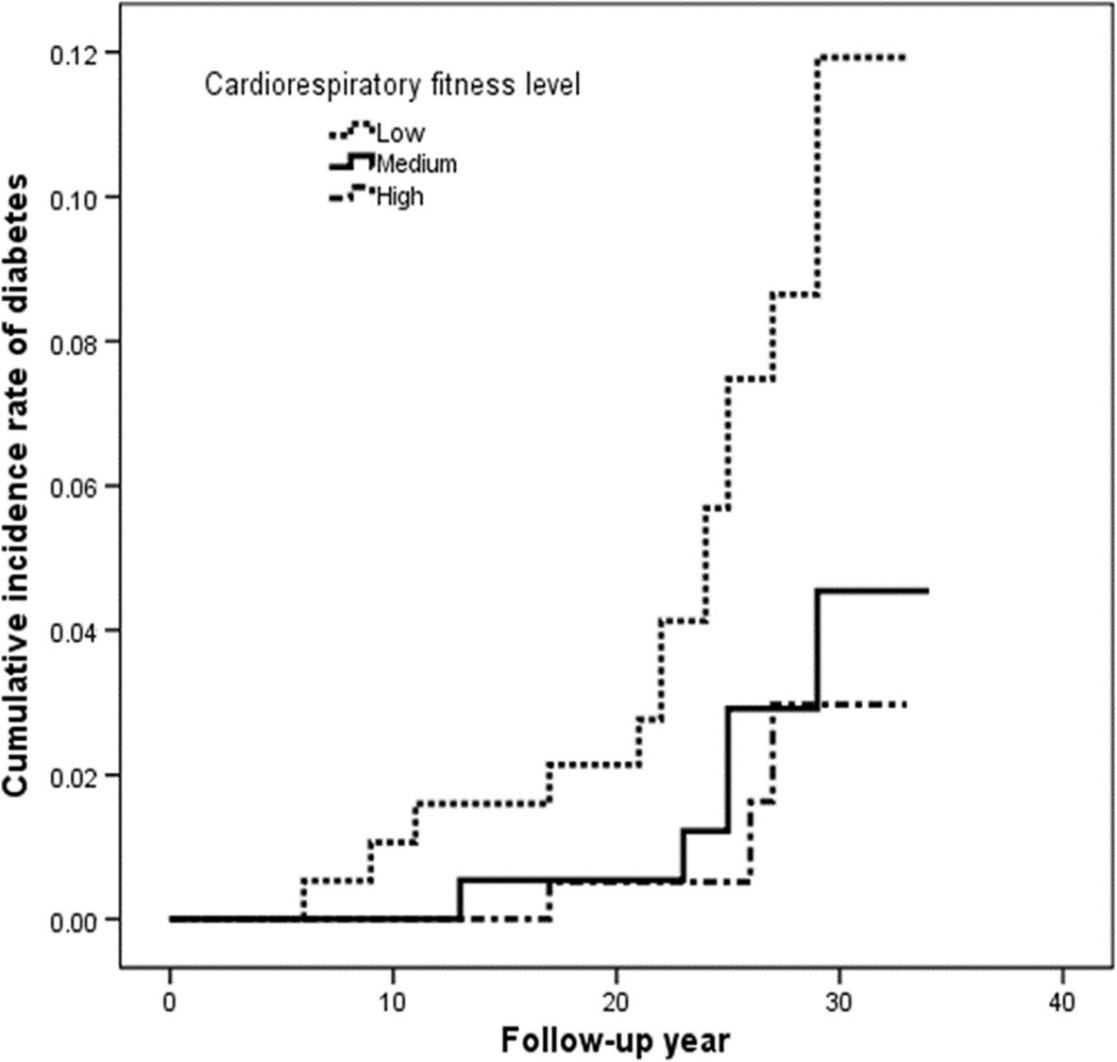
**Cumulative incidence rate curve for type 2 diabetes during follow**-**up**, **according to cardiorespiratory fitness level.**

The incidence of type 2 diabetes per 10,000 person-years was inversely correlated with cardiorespiratory fitness (Table 4). In addition, the low cardiorespiratory fitness level increased the cumulative incidence rate of type 2 diabetes during follow-up (Figure 2). There were progressively lower age-adjusted relative risks of type 2 diabetes across cardiorespiratory fitness levels (p =0.01 for trend). Age-adjusted hazards ratios from low to high were 1.00 (reference), 0.33 (0.12–0.91) and 0.24 (0.07–0.83). After further adjustment for age, year of graduation, BMI, smoking, and college sports club participation, there remained an inverse association between diabetes risk and cardiorespiratory fitness (p =0.03 for trend). The adjusted hazard ratios for the incidence of type 2 diabetes by cardiorespiratory fitness category (low to high) were 1.00 (reference), 0.40 (0.14–1.13), and 0.26 (0.07–1.00). Overall, men in the highest cardiorespiratory fitness group had a 74% lower risk of developing type 2 diabetes than men in the lowest cardiorespiratory fitness group.

**Table 4:** Adjusted hazard ratio for diabetes according to cardiorespiratory fitness level

	**Cardiorespiratory fitness level, tertiles**			**p for Trend**
	**Low**	**Medium**	**High**	
Number of subjects	189	186	195	
Person-years of follow-up	4817	4867	4892	
Diagnosed type 2 diabetes^#^	14	5	3	
Rate per 10,000 parson-years	29.1	10.3	6.1	
Age adjusted hazard ratio (95% CI)	1.00 (Reference)	0.33 (0.12–0.91)	0.24 (0.07–0.83)	0.01
Multivariable adjusted hazard ratio* (95% CI)	1.00 (Reference)	0.40 (0.14–1.13)	0.26 (0.07–1.00)	0.03

*Cox proportional hazards models adjusted for age, year of graduation, BMI, smoking and college sports club participation at college age.

#Diabetes was diagnosed by physician.

## Discussion

In this study, we investigated the relationship between cardiorespiratory fitness at the time of college and the development of type 2 diabetes among Japanese male athletes. Our results show that having a low cardiorespiratory fitness level increased the risk of type 2 diabetes among Japanese male athletes. This result is similar to what had been previously shown among the general Japanese population [7].

To our knowledge, epidemiological studies of fitness and diabetes risk in athletes have previously only been conducted among Caucasian populations. There are reports that former top-level athletes have a low prevalence of type 2 diabetes compared with controls of similar age [8-11]. In particular, former male endurance athletes, such as long-distance runners and cross-country skiers, were shown to have a low prevalence of type 2 diabetes [11]. Although cross-sectional studies have primarily been used to investigate this link, it has been suggested that strength training and high cardiorespiratory fitness specifically contribute to the prevention of type 2 diabetes in athletes.

The Harvard Alumni Study, a long-term cohort study, reported that a low vital capacity at entrance age predisposed subjects to type 2 diabetes later in life [12]. Carnethon et al. also found a significant inverse relationship between cardiorespiratory fitness levels and the incidence of type 2 diabetes in 18- to 30-year-olds [13]. These studies, conducted among Caucasian non-athletes, reported an inverse relationship between cardiorespiratory fitness at a young age and the incidence of type 2 diabetes. The present study indicates that high cardiorespiratory fitness is protective against type 2 diabetes not only in the general population, but also among athletes.

Some plausible mechanisms have been proposed for the link between low cardiorespiratory fitness and diabetes risk; for example, the fact that individuals with low cardiorespiratory fitness tend to have low insulin sensitivity. This positive relationship between the rate of glucose metabolism and maximal oxygen consumption was demonstrated by Sato et al. [14]. Ivy and Kuo also reported that individuals with lower cardiorespiratory fitness levels have fewer glucose transporters than more fit individuals [15].

The cardiorespiratory fitness of athletes is presumably greater than that of the general population. However, the rate of type 2 diabetes in all subjects in the present study was equal to the rate among high cardiorespiratory fitness groups in the Japanese male general population [7]. Therefore, it is appropriate to investigate this topic among athletes. In addition, this was a cohort study of Japanese athletes. Previous studies investigating the relationship between cardiorespiratory fitness and the development of diabetes in athletes were limited to cross-sectional studies and were conducted only among Caucasian populations. Because the incidence of type 2 diabetes differs among ethnic populations; the association of fitness with diabetes risk may also differ among ethnic groups.

Several limitations of this study need to be discussed. First, the subjects are not representative of all Japanese athletes. Only male college alumni were studied. However, these students came from all over Japan, and were selected for admission by an entrance examination of motor skills. In addition, men without both a cardiorespiratory fitness test at college age and a follow-up questionnaire were excluded. These exclusions limit the generalizability of the study, but not its validity. In this study, female alumni were also excluded because they were not registered at the college in 1971. However, we are reflecting on the need to investigate and validate similar data for women. Another limitation is that cardiorespiratory fitness was measured by a 1,500-m endurance run test rather than by laboratory measurements of values such as maximal oxygen consumption. Although the 1,500-m endurance run is a field test, an inverse relationship has been demonstrated between 1,500-m endurance run times and maximal oxygen consumption [16-18]. It was also reported that distance runs over 1 km adequately assess cardiorespiratory capacity [19,20], and that the reliability of field tests has been established for physical education majors [21]. Finally, self-selection bias was possible because the subjects who answered follow-up questionnaires may have been those who were the most healthy. Second recall bias was possible because the questionnaire was cross-sectional and subjects needed to recall their medical background. However, previous studies have used the same method and established its validity [12].

## Conclusions

In conclusion, our results show a strong inverse relationship between the cardiorespiratory fitness of young male athletes and the development of type 2 diabetes later in life. This relationship is independent of age, year of graduation, BMI, smoking, and college sports club participation. We conclude that cardiorespiratory fitness at a young age can predict type 2 diabetes later in life even among Japanese male athletes.

## Competing interests

The authors declare that they have no competing interests.

## Authors’ contributions

YS carried out the interpretation of follow-up data, performed the statistical analysis and drafted the manuscript. SK participated in the design of this study and carried out the follow-up questionnaire research. YK carried out the interpretation of the college including cardiorespiratory fitness. KA participated in the follow-up research and coordination of alumnus. HD conceived of this study and helped to draft the manuscript. All authors read and approved the final manuscript.

## Authors’ information

All authors work at the School of Health and Sports Science, Juntendo University except for HD who is at the School of Medicine, Juntendo University. YS (M.A.) is an assistant whom SK, SK (M.D., Ph.D.) is a professor of sports medicine, YK (Ph.D.) is an assistant professor of measuring evaluation, KA (M.A.) is an associate professor of physical training and HD (M.D., Ph.D.) is a professor of cardiovascular medicine.

## Pre-publication history

The pre-publication history for this paper can be accessed here:

http://www.biomedcentral.com/1471-2458/14/493/prepub
